# Development of Dispersion-Optimized Photonic Crystal Fibers Based on Heavy Metal Oxide Glasses for Broadband Infrared Supercontinuum Generation with Fiber Lasers

**DOI:** 10.3390/s18124127

**Published:** 2018-11-25

**Authors:** Grzegorz Stępniewski, Jacek Pniewski, Dariusz Pysz, Jarosław Cimek, Ryszard Stępień, Mariusz Klimczak, Ryszard Buczyński

**Affiliations:** 1 Faculty of Physics, University of Warsaw, Pasteura 5, 02-093 Warsaw, Poland; grzegorz.stepniewski@gmail.com (G.S.); Ryszard.Buczynski@fuw.edu.pl (R.B.); 2 Department of Glass, Institute of Electronic Materials Technology, Wólczyńska 133, 01-919 Warsaw, Poland; dariusz.pysz@itme.edu.pl (D.P.); jaroslaw.cimek@itme.edu.pl (J.C.); ryszard.stepien@itme.edu.pl (R.S.); mklimcz@gmail.com (M.K.)

**Keywords:** photonic crystal fibers, dispersion, supercontinuum generation, soft glass

## Abstract

In this work a photonic crystal fiber made of a heavy metal oxide glass with optimized dispersion profile is proposed for supercontinuum generation in a broad range of wavelengths in the near-infrared, when pumped by a mode-locked fiber-based laser. The fiber is modelled and optimal geometrical parameters are selected to achieve flat and low dispersion in the anomalous regime. Supercontinuum generation in the range of 0.76–2.40 µm, within the dynamics of 30 dB, when pumped at 1.56 µm with 400 fs–long pulses and an average power 660 mW is possible. The applicability of such fibers is also discussed.

## 1. Introduction

Interest in photonic crystal fibers (PCFs) has lasted for more than two decades since their invention in 1996 [1]. The possibility of dispersion characteristics’ modification by a proper design of the geometrical structure as well as high optical power density in the fiber core led to applications in nonlinear optics [2,3]. Pumping PCFs with high-energy pulses in the anomalous regime of dispersion results in soliton fission that broaden the spectrum of a pulse, due to specific nonlinear phenomena, leading to supercontinuum generation (SG) which can theoretically span several octaves in the frequency domain.

The most popular material of fiber optics, that is fused silica glass, has a relatively low nonlinear refractive index *n*_2_ = 2.74 × 10^−20^ m^2^/W at the wavelength of 1053 nm [4] compared to soft glasses [5] or liquids [6], but its transmission band is limited to approx. 2 μm. The decrease of transmission in silica glass for longer wavelengths, caused by multi-photon absorption by Si–O bondings and vibrational resonances on OH–ions motivates the search for other materials for PCF development that would allow for transmission in mid-infrared (mid-IR). Such transmission is a property of glasses containing heavy atoms with lower excitation energy of optical phonons. A number of these glasses also exhibit higher *n*_2_ than fused silica. For SG a number of glasses are used, such as fluoride glasses, chalcogenide glasses containing compounds of sulphur, arsenic and selenium [7,8,9], tellurite glasses [10] and lead oxide glasses [11,12].

The transmission of ZBLAN glasses ranges up to approximately 5 µm, what allows for SG in the spectral range of 0.8–4.5 µm [13,14], despite relatively low *n*_2_. These glasses have similar linear and nonlinear refractive index to fused silica glass, but their development requires high quality materials that comes at a high cost of the technological process, including the necessity of isolation from external environment to prevent chemical reaction of fluorine compounds with other compounds in the air, and to prevent pollution because of high toxicity of these substances.

Chalcogenide glasses have the largest *n*_2_ ≈ 10^−18^ m^2^/W, among glasses typically used for drawing of fibers, and the zero dispersion wavelength (ZDW) is located in mid-IR (*λ*_ZDW_ > 4.5 µm), while the transmission wavelength range extends to over a dozen of micrometers, depending on chemical composition. Pumping of fibers made of these glasses in the anomalous dispersion regime (*λ_p_* > *λ*_ZDW_) requires advanced mode-locked lasers tuned in the range of 4–7 μm. In the paper by Petersen et al. [8] SG in the range of 1.4–13.3 µm was demonstrated in step-index chalcogenide fibers, under pumping with 100 fs–long pulses, centered at a wavelength of *λ_p_* = 6.3 µm and with peak power of *P*_0_ = 2.3 MW, from a complex optical parametric amplifier system.

Zhao et al. also demonstrated mid-IR SG in a low-loss Te-based chalcogenide step-index fiber [15]. The fiber exhibits an optical loss of 2–3 dB/m in the range of 6.2–10.3 μm and 3.2 dB/m at 10.6 μm. The supercontinuum spectrum was generated in the range of 1.5–14 μm when a 23 cm–long fiber was pumped by a 4.5 μm laser with 150 fs–long pulses and the repetition rate *f_rep_* = 1 kHz.

Recently, mid-IR SG has been demonstrated in a low-loss telluride glass fiber with a double cladding. The fiber has attenuation lower than 10 dB/m in the range of 8–14 μm. Ultrashort pulses with a central wavelength of 7 μm, allowed for SG spanning from 2.0 μm to 16 μm for a 40-dB spectral flatness [16].

Furthermore, a suspended–core PCF made of tellurite glasses (tellurium oxide glass) has been demonstrated by Domachuk et al. [17] that enable SG in the range of 0.79–4.87 µm. The core diameter was 2.5 µm, while *λ*_ZDW_ = 1.38 µm. The fiber was pumped using an optical parametric oscillator (OPO) emitting 110 fs–long pulses with *f_rep_* = 80 MHz for *λ_p_* = 1.55 µm and the mean power *P*_avg_ = 150 mW. Less than 1 cm of fiber was used, which was enough to generate hyperspectral supercontinuum at a standard pump wavelength, due to high nonlinearity of the fiber and ultrashort pump pulse duration. It also allowed to broaden the supercontinuum spectrum over 3 µm despite usually high absorption of multicomponent oxide glasses at this wavelength due to OH impurities. On the other hand, telluride glasses are fragile what makes development in the low-cost stack-and-draw process problematic. The suspended core geometry does allow high nonlinearity due to strong confinement, but at the same time it is also strongly isolated thermally from the cladding that leads to a fiber being more susceptible to laser damage, compared to other types of PCFs with low air-glass filling factor.

PCFs made from heavy metal oxide glasses, such as SF_6_ with *n*_2_ = 2.2 × 10^−19^ m^2^/W, are an alternative to tellurite glasses. Omenetto et al. [11] demonstrated a fiber with four air-holes surrounding a core of diameter *d_r_* = 2.6 µm, which exhibited *λ*_ZDW_ = 1.30 µm. When pumped with an OPO source with *λ_p_* = 1.55 µm SG was achieved in the range of 0.7–3.0 µm with a dynamics of 40 dBs.

Other glasses are also used, e.g. bismuth-based glasses. In one of our previous works, we have demonstrated SG in the range of 100–2500 nm with a 5 dB flatness in a PCF made of lead-bismuth-gallate glass and pumped in the femtosecond regime at 1540 nm [18]. Later, a PCF which allowed for SG in the range of 1.2–2.0 µm was shown, when pumped with OPO source with *λ_p_* = 1.55 µm, 100 fs–long pulses, and *f_rep_* = 1.0 kHz [19]. Finally, spectral and coherence evolution were experimentally measured for SC generated in PCFs made of SF_6_ glass [20]. A significant part of our previous work in multi-component oxide soft glass was related to fibers with normal dispersion profiles and a comprehensive review of coherent supercontinuum generation in soft glass photonic crystal fibers can be found in [21]. Table 1 summarizes selected typical properties of glasses used for development of PCFs.

Using OPO sources for SG does not allow for building compact all-fiber systems with broad and high power emission spectrum in the near- and mid-IR, which is important, e.g., in fluorescent microscopy [26], optical coherence tomography [27] and environmental monitoring [28].

In this work we analyze the performance of a PCF made of a heavy metal oxide glass that allows for SG over a full octave under pumping from mode-locked, fiber-based lasers emitting pulses at 1.56 μm. First, the fiber is modelled and optimal geometrical parameters are selected to achieve flat and low chromatic dispersion in the anomalous regime for spectrally efficient, soliton-based SG. Then, the fiber is developed in the stack-and-draw process and, finally, its nonlinear performance is investigated using SG simulations and physical experiment.

## 2. Numerical Simulations of Linear Properties of the PCF

**Figure 1:**
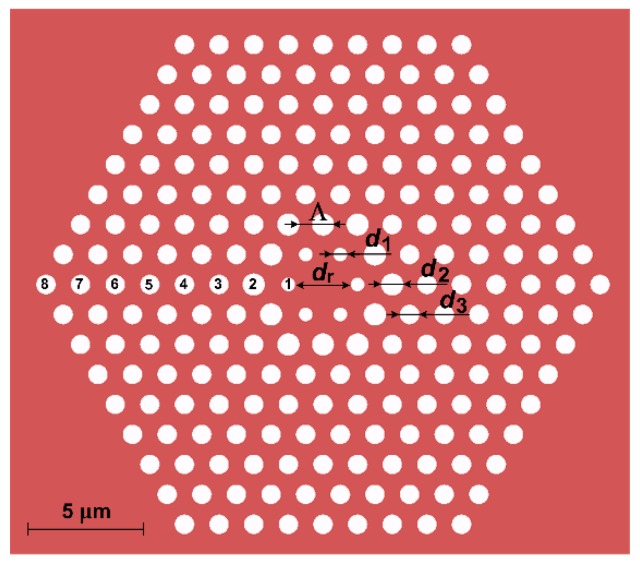
The schematic of the structure of the analyzed PCF. The numbers from 1 to 8 represent subsequent rows of holes surrounding the core.

The analyzed PCF consists of a solid core surrounded by a hexagonal air-hole photonic cladding. Its structure is shown in Figure 1, where *d_r_* denotes the diameter of the core, *d*_1_—the diameter of the holes directly surrounding the core in the first row, *d*_2_—the diameter of the holes in the second row, and *d*_3_—the diameter of the remaining holes. Due to variable diameters of the holes this structure holds effectively three different filling factors *d*/*Λ* in the cladding.

**Figure 2:**
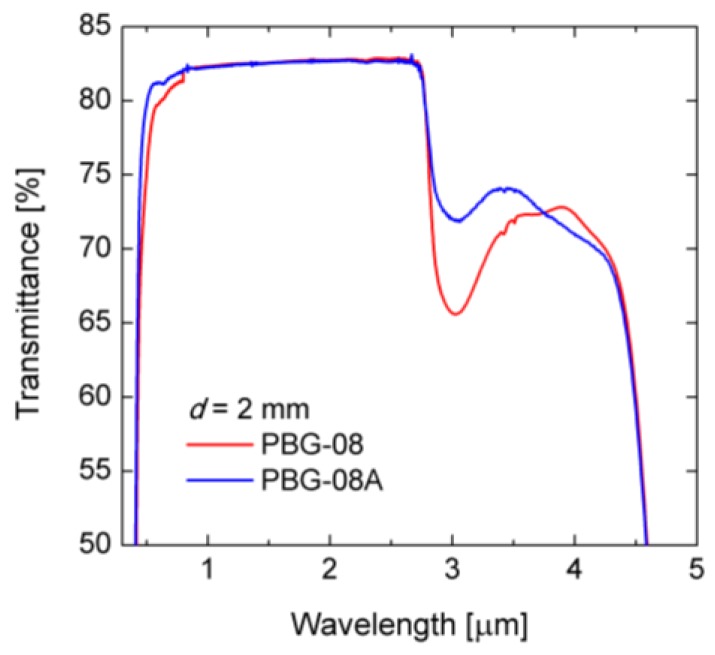
Characteristics of transmission for PBG-08 and PBG-08A glasses, for a 2 mm–thick sample.

As a base material for the PCF PBG-08A glass was chosen. This is an in-house developed glass with the chemical composition similar with that of the lead-bismuth-galate PBG-08 glass [18]. The glass was developed under a protective gas atmosphere, what led to the reduction of the water content. As a result, the transmission of the glass was increased in comparison with PBG-08 to 75% for the wavelength of 3 μm. The characteristics of the transmission for both glasses are shown in Figure 2.

The refractive indices of PBG-08 and PBG-08A glass are similar but *n*_PBG-08A_ is higher than *n*_PBG-08_ by approx. 0.005. The measurement was done using a Michelson interferometer with the accuracy of 0.002. The refractive index characteristic of PBG-08A glass is modelled using Sellmeier’s equation below. The Sellmeier’s coefficients calculated on the basis of experimental results are presented in Table 2.

(1) $$n\left(\lambda \right)=\sqrt{1+\frac{B_{1}\lambda^{2}}{\lambda^{2}-C_{1}}+\frac{B_{2}\lambda^{2}}{\lambda^{2}-C_{2}+}+\frac{B_{3}\lambda^{2}}{\lambda^{2}-C_{3}}}.$$

The aim of the numerical study was to design a new air–glass structure of a photonic cladding that allows for single mode operation and for flat and low chromatic dispersion in the anomalous regime. We analyzed the influence of the geometrical parameters of the structure, namely the lattice constant *Λ*, and diameters of the holes, *d*_1_, *d*_2_, *d*_3_, on linear properties of the PCF in terms of effective refractive index *n_eff_*(*λ*), attenuation *A*(*λ*), and dispersion *D*(*λ*) for the wavelength range of 0.8–5 μm. We assumed that the PCF would be coupled to a mode-locked laser emitting at *λ_p_* = 1.56 µm, therefore ZDW should be lower than the pump wavelength. A commercial-grade simulator, eigenmode solver and propagator was used to perform the calculations [29].

**Figure 3:**
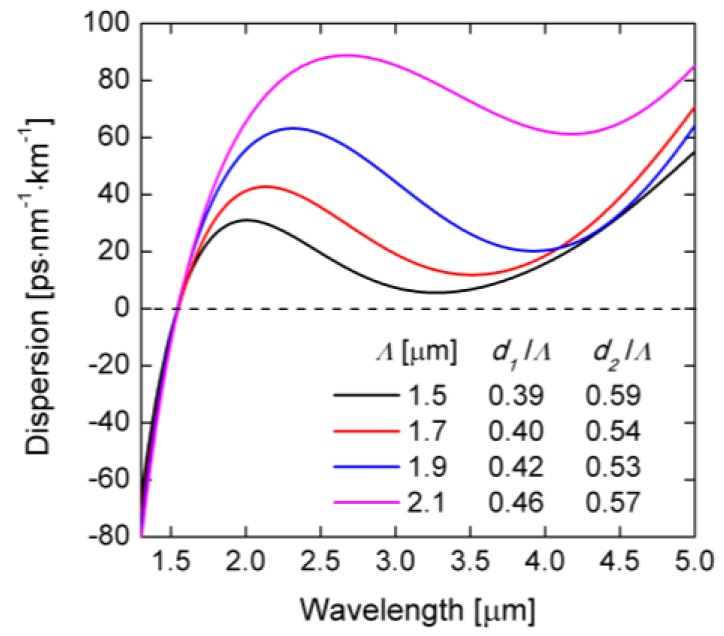
Dispersion characteristics for different lattice constants *Λ* and different filling factors for the first row of air-holes *d*_1_/*Λ*, and for the other rows of air-holes *d*_2_/*Λ*.

The possibility of controlling the lattice constant *Λ* and the diameter of air-holes in three subsequent rows allows for precise shaping of dispersion characteristics in a wide range of wavelengths. First, the influence of the lattice constant was simulated for *Λ* in the range of 1.5–2.1 µm and two different filling factors in the first and outer rows of holes. The resulting dispersion characteristics of the fundamental mode are shown in Figure 3. The local maximum of the dispersion characteristic in the vicinity of ZDW is the lowest for *Λ* = 1.5 µm. Thus, this value was used for further analysis.

**Figure 4:**
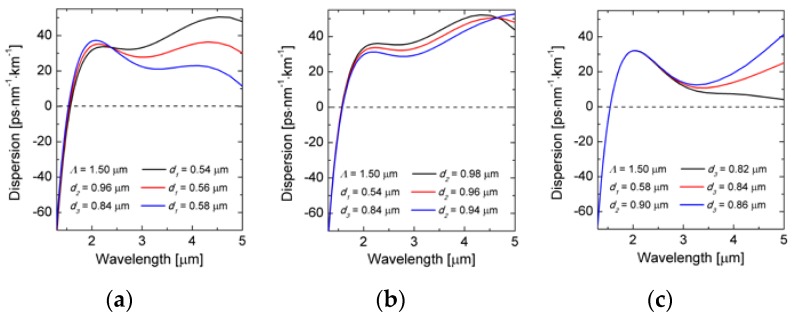
Dispersion characteristics for the lattice constant *Λ* = 1.5 μm and varied diameters of air-holes in (**a**) the first row *d*_1_, (**b**) the second row *d*_2_, and (**c**) the other rows of the photonic cladding *d*_3_.

The subsequent computations were performed for a set of diameters *d*_1_, *d*_2_ and *d*_3_. Selected dispersion characteristics are shown in Figure 4. The change of the diameter *d*_1_ has the biggest influence on the location of ZDW and the shape of the dispersion characteristic in the range of 1.0–5.0 μm (see Figure 4a) [30]. The change of the diameter *d*_2_ has only small effect on ZDW and influences the dispersion characteristic mainly in the range of 2–4.5 μm (see Figure 4b). The change of the diameter *d*_3_ has almost no effect on ZDW but influences the dispersion characteristic in the range of 2.5–5 μm (see Figure 4c). Thus, using three different filling factors in the lattice geometry, one has the opportunity to precisely shape the dispersion characteristics, but at the same time even small variations in the diameters of the air-holes during fiber development, on the level of tens of nanometers, can lead to a significant change of the characteristics.

**Figure 5:**
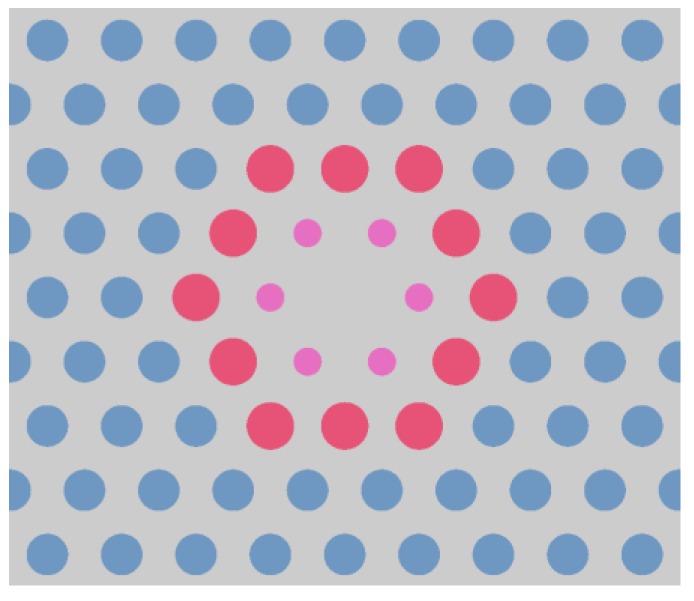
The close-up of the final dispersion-optimized structure. The diameters of the holes are in scale.

**Figure 6:**
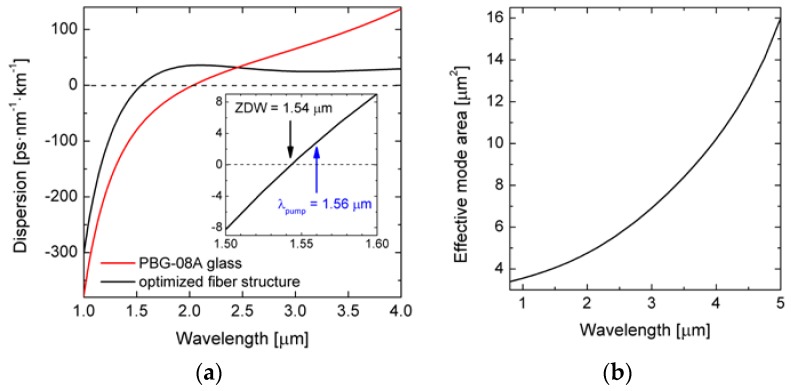
(**a**) Optimal dispersion characteristic and (**b**) effective mode area of the dispersion-optimized PCF.

As a final dispersion-optimized structure the fiber with parameters given in Table 3 was chosen, that exhibits the lowest and flat dispersion in the vicinity of ZDW in the anomalous dispersion regime. The geometrical structure of this PCF is shown in Figure 5. The dispersion characteristics and the effective mode area *A*_eff_ are shown in Figure 6. In this fiber ZDW is located at 1.543 µm and the maximum dispersion value *D*_max_ = 36 ps/(nm·km) was obtained for the wavelength of 2.106 µm. For *λ_p_* = 1.56 µm the total fiber dispersion equals *D* = 2.8 ps/(nm·km) while the group dispersion equals *β*_2_ = −3.6 ps^2^/km and calculated *A*_eff_ is 4.13 μm^2^.

**Figure 7:**
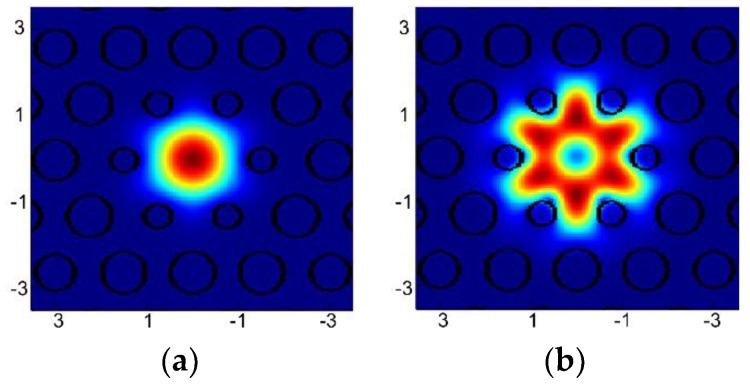
Numerically simulated distributions of intensity of modes for the wavelength of 1.56 µm: (**a**) fundamental mode LP_01_, (**b**) mode LP_31_.

**Figure 8:**
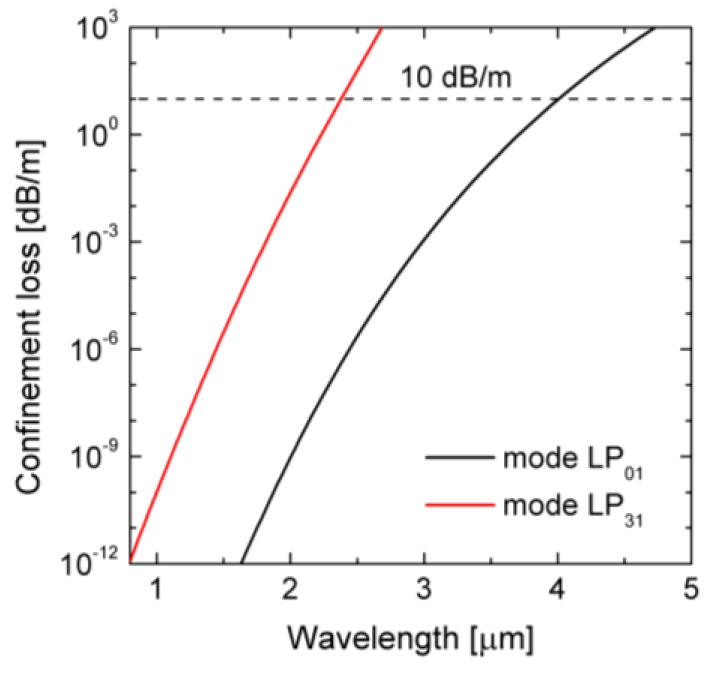
Spectral characteristics of attenuation for propagating modes LP_01_ and LP_31_.

In the next step, the mode analysis for the dispersion-optimized PCF was performed. Two-dimensional distributions of intensity of the only propagating modes are shown in Figure 7. Additionally, attenuation was computed for the analyzed modes in the range of 0.8–5.0 μm, shown in Figure 8. As a cut-off attenuation criterion 10 dB/m was selected [31]. Therefore, propagation of the mode LP_31_ is possible, but its attenuation is higher than attenuation of the fundamental mode (FM) by a factor of 10^9^. Theoretical cut-off wavelength for the mode LP_31_ is 2.38 μm and for the FM it is 4.0 μm, which means that the fiber does not guide light in the core for wavelengths longer than 4.0 μm. Although for the pump wavelength *Λ_p_* = 1.56 µm two modes are guided, the effective coupling efficiency is equal 0.3 for FM and close to zero for the mode LP_31_, when a Gaussian beam with numerical aperture 0.6 is considered.

## 3. PCF Development

**Figure 9:**
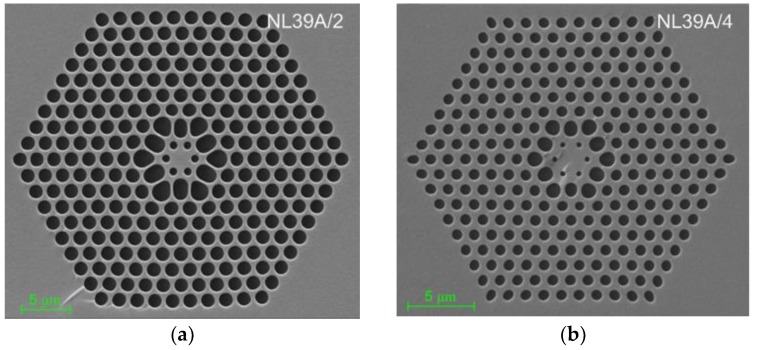
SEM images of the cross-sections of the developed fibers (**a**) #A2 and (**b**) #A4.

Dispersion-optimized PCFs were then developed in a stack-and-draw process [32]. The drawing process had two stages. First, a subpreform was stacked from capillary tubes and rods and the initial drawing was done with the low pressure to close the gaps between capillaries and rods. Capillaries had different internal diameters for the particular rows of air-holes. Then, the top of the resulting preform was sealed and the final fiber was drawn. The increase of the pressure in the capillaries caused by the increase of the temperature influenced the diameters of the holes. A series of PCFs was drawn, using a prepared subpreform, which differ in size and internal structure. The differences are a result of different drawing speed. The images of the cross-sections of two selected fibers with structures close to the designed one, labeled #A2 and #A4, made by a scanning electron microscope, are shown in Figure 9. The geometrical parameters of PCFs are presented in Table 4, where the external diameter of the fiber is denoted as *Ø*_out_.

The structures of PCFs are different from the ideal one but at the same time they are stable over the lengths of single-digit meters, while for SG only tens of centimeters are required. It means that having a certain developed structure its performance is not degraded due to fabrication errors. Stack-and-draw technology does not allow for such precise control of air-hole sizes even if we close the top end of the preform [33,34]. For technological reasons we were not able to use different pressure for subsequent rows of air-holes as suggested in [35,36], but there are a number of factors influencing the final structure, including velocity of drawing, temperature distribution in the furnace, length of the furnace, thickness of the fiber, localization of air-holes in the cladding. Nevertheless, the shape and symmetry of the cladding is maintained. 

The air-holes in the second row are deformed and have oval or elliptical shape. The lattice constant is not preserved in the whole cladding and is reduced in the first ring of holes due to large diameter of the air-holes in the second row. For the fiber #A2 the lattice constant decreases from approx. 1.8 µm to 1.4 µm, while for the fiber #A4 from approx. 1.3 µm to 1.2 µm. The fiber #A2 has significantly bigger filling factor in the rows from 2 to 9 than in case of the designed structure. This discrepancy is decreased in the fiber #A4 through the reduction of the diameters of the air-holes that, in turn, led to the decrease of the diameters of the air-holes surrounding the core.

## 4. Characterization of Linear Properties of Developed PCFs

The developed fibers were characterized with respect to linear optical properties, namely mode structure, attenuation, numerical aperture (NA) and chromatic dispersion. The modes were determined at the wavelength of 1.56 µm using an amplified spontaneous emission source (ASE), capable to emit spatially coherent light in the range of 1.51–1.62 µm. The output plane of the PCF was imaged on a phosphorus-enriched CCD camera using a microscopic objective 60×/0.85, what allowed to record IR images in the range of 1.46–1.60 µm.

A change of the position of the fiber with respect to the focus of the light-introducing lens was applied to excite the fundamental and higher order modes. The measurement was performed for both long (more than 2 m) and short (40–50 cm) sections of the fibers. For long sections only fundamental mode was observed. 

**Figure 10:**
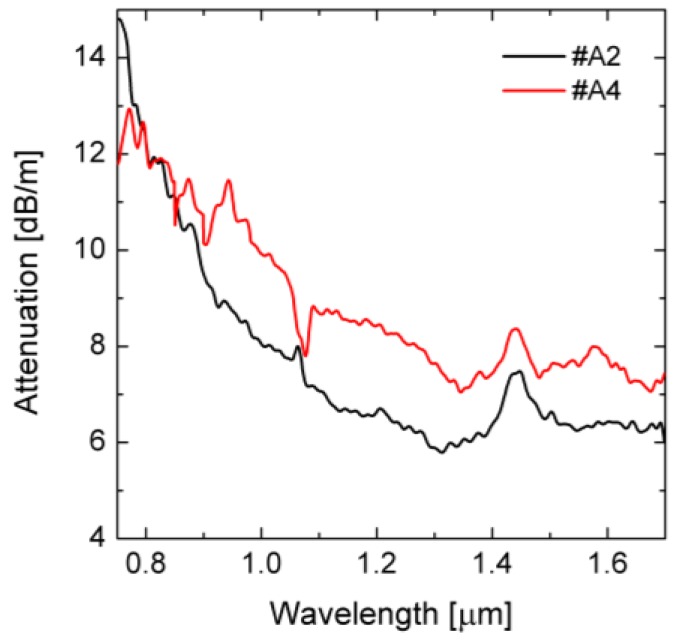
Measured attenuation characteristics for the fibers #A2 and #A4.

The attenuation was measured using the cut-back method. The resulting attenuation characteristics are shown in Figure 10. The characteristics are typical for the PBG glasses with the increase below 1.30 µm and the peak of 7–10 dB/m around 1.55 µm. For *λ_p_* = 1.56 µm the attenuation reaches 6.3 dB/m and 7.7 dB/m for the fibers #A2 and #A4, respectively. Higher attenuation of the fiber #A4 compared to #A2 results from higher confinement losses and fabrication imperfections. The latter causes an emission of light to the photonic cladding and outer glass layer through micro-ruptures that are common in soft glasses.

The numerical apertures of the fibers #A2 and #A4 were measured with ASE source with central wavelength of 1.56 µm using the CCD camera and under the assumption that the beam is Gaussian with the waist located at the end of the fiber. For the fiber #A2 NA = 0.52, while for the fiber #A4 NA = 0.51.

**Figure 11:**
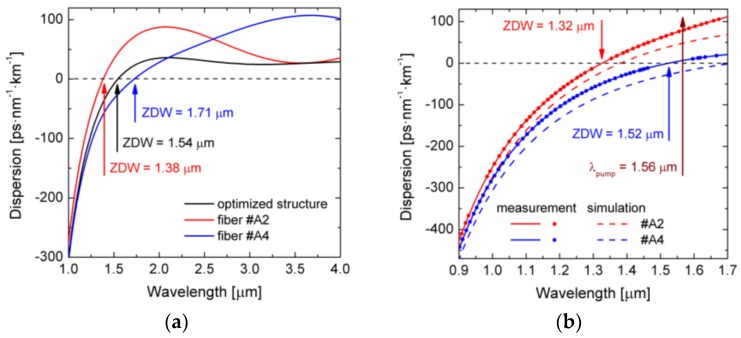
Dispersion characteristics of the fibers (**a**) #A2 and #A4 (simulation) compared to the dispersion characteristic of the ideal dispersion-optimized structure, (**b**) A2 and #A4 (both simulated and measured).

The chromatic dispersion was both numerically modelled and experimentally measured. Simulations were performed on the basis of SEM images of the fibers, while for the measurement we used a Mach-Zehnder interferometer [37]. The resulting dispersion characteristics are shown in Figure 11 and the values of ZDW and the dispersion *D_p_* for pumping at *λ_p_* = 1.56 µm are presented in Table 5. The dispersion of the fiber #A2 for *λ_p_* is located in the anomalous regime. In case of the fiber #A4 ZDW is located close to *λ_p_* and actually the fiber can be excited in the normal or anomalous regime.

Measured and predicted in numerical simulations chromatic dispersion characteristics do not overlap, although the overall tendency is maintained. Measured values are shifted towards shorter wavelengths. In case of the fiber #A4 the wavelength difference equals 189 µm. There are a number of factors influencing the outcome of the numerical simulations. First, the procedure of binarization of the SEM image may not be accurate and, thus, change the diameters of the air-holes. Second, the structure of the PCF along the fiber is not perfectly maintained. As a result, the actually measured fiber can be different from the fiber imaged by SEM. Third, the accuracy of the Mach-Zehnder interferometer is limited to single-digit nanometers.

The effective mode are was modelled on the basis of SEM images. For the fiber #A2 *A*_eff_ = 3.11 µm^2^, while for the fiber #A4 *A*_eff_ = 3.70 µm^2^. 

## 5. Supercontinuum Generation in Developed PCFs

Simulations of SG were performed for both the dispersion-optimized ideal structure and the developed fibers #A2 and #A4. Generalized nonlinear Schrödinger equation (GNLSE) was solved numerically to simulate the pump pulse propagation, and specifically the split-step Fourier algorithm was implemented [38,39]. In the GNLSE of the form of:

(2) $$\;\frac{\partial A}{\partial z}+\frac{\propto }{2}A-\underset{k\geq 2}{\sum }\frac{i^{k+1}}{k!}\beta_{k}\frac{\partial^{k}A}{\partial T_{k}}=i\gamma \left(1+i\tau_{shock}\frac{\partial }{\partial T}\right):\times \left(A\left(z,T\right)\overset{+\infty }{\underset{-\infty }{\int }}R\left(T^{\prime }\right)\left|A{\left(z,\;T-T^{\prime }\right)}^{2}\right|dT^{\prime }\right)\;$$

where *A* represents the complex pulse envelope, *z* is the coordinate along the fiber (propagation) length, and the left-hand side describes linear processes, while the right-hand side describes the nonlinear processes. The second term in the left-hand side accounts for a linear loss with loss coefficient *α*, and the third term represents the dispersion with the dispersion coefficient *β_k_* associated with Taylor series expansion of the propagation constant *β*(*ω*) about central frequency *ω*_0_. At the right-hand side, the nonlinear coefficient is given by *γ* = n_2_∙ω_0_/(*c*∙*A*_eff_), where *n*_2_ = 2 × 10^−19^ m^2^/W is the nonlinear refractive index of the fiber glass, here measured using z-scan at 1064 nm [5], the Raman response of glass is parametrized analogically to [38,39], and specifically the first order Raman shift frequency for the fiber glass in this work is *Ω_R_* = ±29 THz, and the Lorentzian fit to the first-order Raman scattering term is described by time-frequency of excited optical fonons *τ*_1_ = 5.5 fs, and time-width of Lorentz band *τ*_2_ = 32 fs. The contribution of delayed Raman scattering response to the Kerr nonlinearity was *f_R_* = 0.05. The simulation time window corresponded in the spectral domain to the wavelength range of 0.8–5.0 μm. The pump pulse duration (auto-correlation width) was *t_imp_* = 400 fs. 

**Figure 12:**
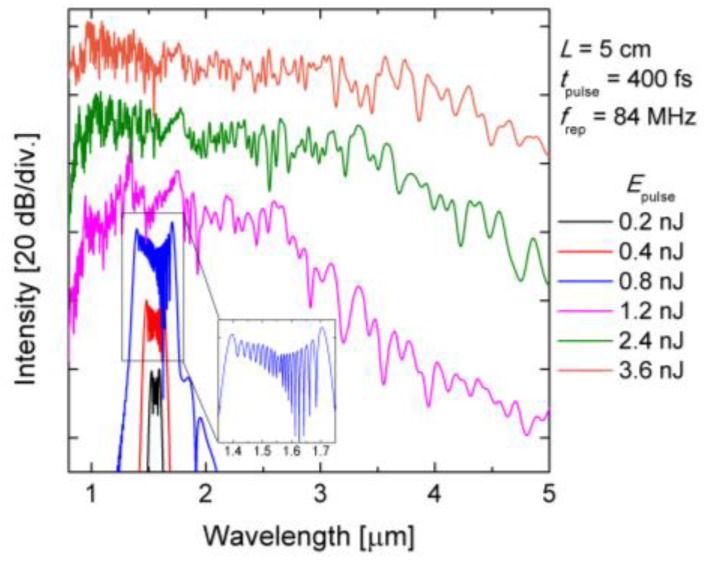
Numerical simulation of SG in the dispersion-optimized PCF, for pulse energy in the range of 0.2–3.6 nJ. For clarity characteristics are separated vertically by 20 dB.

In Figure 12 supercontinuum pulse spectra simulated for the ideal dispersion-optimized PCF are shown for the wavelength range of 0.8–5.0 μm, for different input pulse energies. The length of the fiber is 5 cm. For the pulse energy *E_imp_* = 0.8 nJ the broadening dynamics are limited practically to self-phase modulation (SPM) within a wavelength span of approximately 400 nm and within 20 dB dynamic range. Increasing *E_imp_* to more than 1 nJ results in further spectrum broadening, and for *E_imp_* = 2.4 nJ supercontinuum goes up to 3.6 μm in IR within 20 dB dynamics. In Table 6 dispersion lengthscale *L_D_*, nonlinear lengthscale *L_NL_*, and soliton fission lengthscale *L_sol_* for *N-*order solitons are summarized, for the pulse energy *E_imp_* = 2.4 nJ. The dispersion lengthscale is expressed as *L_D_* = *t*_0_^2^/|*β*_2_|, while the other lengthscales are defined as *L_NL_* = 1/*γP*_0_ (where *P*_0_ is the peak pump power), *L_sol_* = *L_D_*/*N*, and the soliton order is expressed as *N* = *L_D_*/*L_NL_*. 

**Figure 13:**
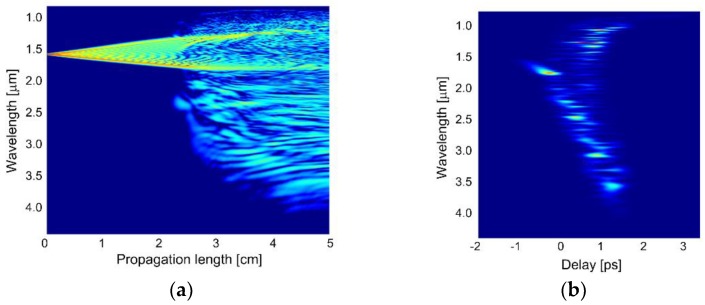
(**a**) Evolution of the pulse and (**b**) spectrogram for the dispersion-optimized PCF.

The evolution of the pulse as a function of the propagation length is shown in Figure 13a, while the calculated group delay trace (spectrogram) is shown in Figure 13b, for *E_imp_* = 2.4 nJ and *L* = 5 cm. At the beginning of the fiber, the spectrum of the pulses expands due to SPM. This is expected, because *L_NL_* is larger than *L_D_*, therefore SG occurs with dominance of nonlinear contribution over dispersion. Around 2 cm of propagation, the high order input soliton has already undergone fission into multiple low order solitons, which is in rough agreement with the 1.3 cm value estimated using formula *L_sol_* = *L_D_*/*N*. Due to high order *N* = 334 of the soliton introduced by the pump, the supercontinuum is expected to be time-incoherent, despite femtosecond pumping [40]. The modulation instability lengthscale can be estimated as 16∙*L_NL_*, which in this case is just over 6 cm. This result provides rationale for assuming the fiber length of 5 cm, in order to observe supercontinuum generation before amplification of significant noise. Soliton fission and their subsequent Raman redshift is accompanied by a radiation of higher frequencies, the dispersive waves, which is responsible for broadening of the supercontinuum spectrum towards shorter wavelengths. As a result of the decay the lower-order solitons are shifted towards IR due to Raman scattering (SSFS, soliton self-frequency shift). Because of relatively low anomalous chromatic dispersion at wavelengths redshifted from ZDW (0–36 ps/(nm·km)) broad phase matching can be achieved in the range of 1–4 μm for the degenerated four-wave mixing.

**Figure 14:**
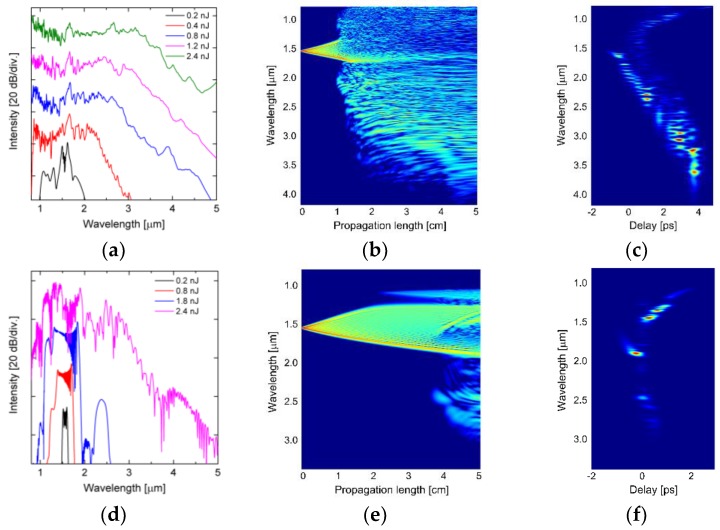
Numerically generated (**a**,**d**) supercontinuum spectra for in the range of 0.2–2.4 nJ, (**b**,**e**) evolution of the spectra as a function of the length of the fiber for *E_imp_* = 2.4 nJ, (**c**,**f**) time–delay spectrograms. The upper rower depicts the fiber #A2, while lower—#A4.

In the next step, nonlinear propagation simulations were conducted using fiber linear properties data calculated for real fiber structures using SEM images of the fibers #A2 and #A4. The results are shown in Figure 14, while in Table 7 nonlinear parameters are shown, namely group velocity dispersion *β*_2_, third-order dispersion *β*_3_, nonlinear coefficient *γ*, dispersion length *L_D_,* nonlinear length *L_NL_*, soliton decay length *L_sol_*, order of solitons *N*, for *λ_p_* = 1.56 µm and *E_imp_* = 2.4 nJ. 

In the nonlinear propagation simulations for the fiber #A2, it is assumed that pumping falls into wavelengths, where there is anomalous chromatic dispersion. Results of simulations are summarized in Figure 14a–c. High nonlinearity results in pulse nonlinear lengthscale of *L_NL_* = 0.3 mm, however *L_D_* is also short, one order of magnitude larger than *L_NL_*, as opposed to two orders of magnitude in the ideal fiber. First 1 cm of propagation is dominated by SPM, which is followed by soliton fission and Raman redshift, accompanied by dispersive wave generation. Calculated soliton order is over three times smaller, than in the fiber considered in the design stage, thus the group delay trace shows several discernible soliton features between around 0.5 and 4 ps of delay (Figure 14c). Due to very short nonlinear length scale, the estimated modulation instability lengthscale is just below 5 cm, suggesting onset of noise amplification and decoherence of supercontinuum pulses over the final 2 mm of the fiber. The width of the spectrum is 0.8–3.7 μm within 20 dB dynamics.

In the nonlinear propagation simulations for the fiber #A4, the *λ_ZDW_* > *λ_p_* and the pump pulse is introduced at wavelengths with normal dispersion regime. The broadening is again initiated by SPM, because *L_NL_* is still roughly 20 times shorter than *L_D_*. When the redshifted part of SPM reaches and crosses ZDW at 1.714 µm, solitons begin to emerge at anomalous dispersion wavelengths. Their redshift is faintly visible in the group delay trace in Figure 14f just before end of 5 cm of propagation. In this scenario, the redshifting solitons also generate dispersive waves across ZDW, which here result in destructive beating with the normal dispersion-broadened spectral components, giving rise to complicated spectral evolution both in the group delay (Figure 14f) and along the fiber length (Figure 14e). The supercontinuum spectrum covers 1.0–2.9 μm.

**Figure 15:**
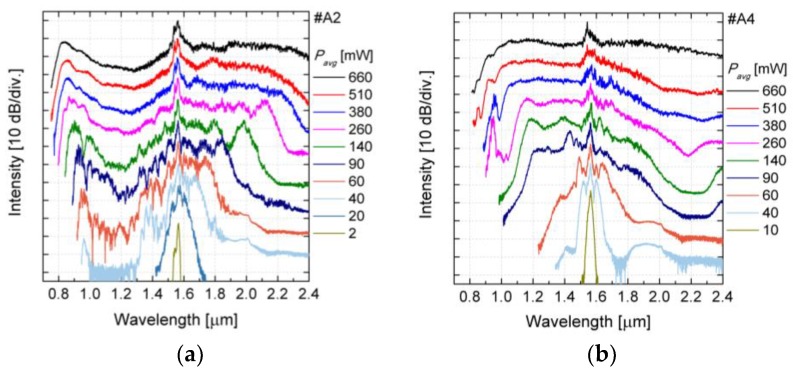
Measured supercontinuum spectra for the fibers (**a**) #A2 and (**b**) #A4 for different average power of the pumping laser. For clarity characteristics are separated vertically by 20 dB.

For the coupling of pump laser light to the fibers in the experimental part of work, two microscopic objectives 40×/0.65 were used. At the output, the collimated beam was focused on a front face of a multi-mode fiber, using an achromatic lens with *f* = 50 mm. The multi-mode fiber was connected to an optical spectrum analyzer (OSA). Two OSAs were used, both from Yokogawa, one device covered spectral range of 0.6–1.7 µm and the other of 1.2–2.4 µm. The length of both nonlinear PCFs (#A2 and #A4) was *L* = 15 cm and the coupling efficiency was estimated at 15%. The length of the fiber samples was motivated with handling convenience. The broadening of the spectrum as a function of average power *P_avg_* of the laser source is shown in Figure 15. 

Taking into account the coupling efficiency approx. *P_avg_* = 100 mW was obtained, which corresponded to peak power of *P*_0_ = 2.95 kW and coupled pulse energy of *E_imp_* = 1.18 nJ. In the physical experiments with the fiber #A2, pumping is in the anomalous dispersion wavelengths, and the spectrum is efficiently broadened towards the short-wave range. For *P_avg_* = 60 mW (*E_imp_* = 0.11 nJ) a peak appears for the wavelength of 0.96 μm caused by the dispersive wave caused by the soliton decay in the long-wavelength range. At this pump power the spectrum is still non-uniform and exhibits a decrease of the intensity in the range of 0.96–1.4 μm along with the spectrum limit at 1.80 μm. The increase of the pump power results in the broadening of the spectrum towards IR. For wavelengths shorter than *λ_p_* the spectrum becomes smooth and flat and the short-wavelength limit is shifted towards visible wavelengths but slower than long-wavelength limit. For *P_avg_* = 660 mW (*E_imp_* = 1.18 nJ) the spectrum still shows a clearly pronounced dispersive wave separated by a slightly less intense plateau in the range of 0.84–1.51 μm wavelengths. For the dynamic range of 30 dB the spectral coverage of SG in fiber #A2 is not less than 0.76–2.4 μm wavelengths, since the detection limit of OSA was 2.4 μm.

The fiber #A4 is pumped very close to the ZDW, and part of the pump pulse spectrum falls into normal dispersion wavelengths, while part of the pulse spectrum covers anomalous dispersion wavelengths of the fiber. For average power of *P_avg_* = 60 mW the pulse is broadened only due to SPM and its width is equal to 0.41 μm (1.35–1.76 μm) within 30 dB dynamics. The increase of the power of the laser in the range of 90–510 mW broadens the flat supercontinuum spectrum in the short-wavelength range. For waves longer than 1.56 μm the intensity decreases with the wavelength. Starting from *P_avg_* = 260 mW a blue-shifted peak appears in the spectrum at about 0.9 μm that is assigned to a dispersive wave related to a soliton, which emerges at the anomalous dispersion side of the ZDW, where enough energy is transferred over to the anomalous dispersion wavelength range of fiber. For the maximum available pump power, the supercontinuum covers wavelength range of 0.86–2.4 μm (measurement limited by OSA sensitivity range). This less reach in terms of blue-shifted wavelength than the width for the fiber #A2, but at the same time the spectrum is flatter than in the case of #A2 fiber.

**Figure 16:**
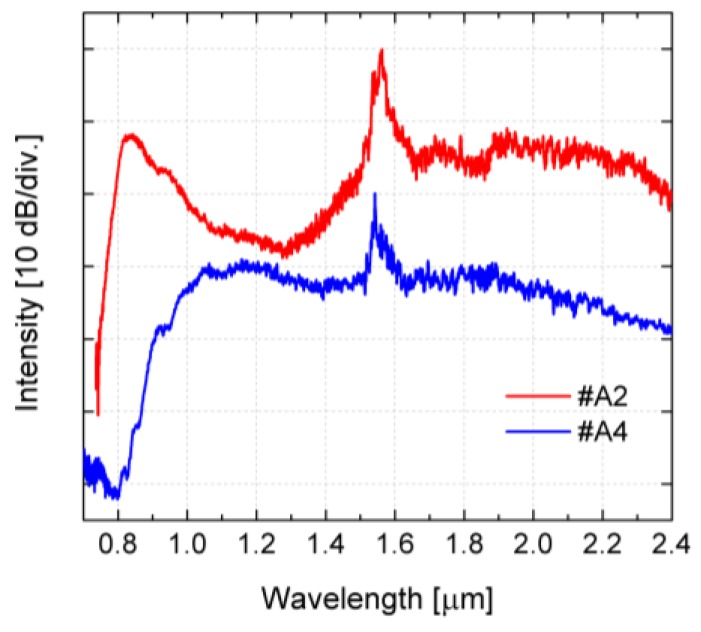
Measured supercontinuum spectra for the fibers #A2 and #A4 and *P_avg_* = 660 mW. For clarity characteristics are separated vertically by 20 dB.

In Figure 16 the characteristics of supercontinuum spectrum are compared, for the fibers #A2 and #A4, for *P_avg_* = 660 mW. The width of supercontinuum spectra, for different dynamics and the length of the fibers 15 cm, are shown in Table 8.

## 6. Conclusions

In the paper the possibility to shape the dispersion characteristics of a PCF due to a precise choice of the diameters of the air-holes in the cladding, including variable filling factor, was demonstrated in the fiber made of a heavy metal oxide glass PBG-08A. The influence of certain rows of holes surrounding the core on the dispersion characteristics was determined. Then, a proof-of-concept was shown, including two physically developed fibers with designed parameters, developed in the stack-and-draw process. It was proven, both in simulations and experimentally, that supercontinuum generation over broad wavelength range in the near-IR with this PCF is possible, when the fibers are pumped with a standard fiber-based laser wavelength of 1.56 µm with 400 fs–long pulses with an average power 660 mW.

The presented PCF is well suited to all-fiber systems, and does not require OPO for pumping. The proof-of-concept also evidenced that good control of the technological development process is crucial for the repeatability of the fiber’s structure. Using variable filling factor in the lattice geometry of the photonic cladding allows for dispersion engineering in a broad range, but at the same time a small change of a diameter of air-holes in one row influences the lattice constant, the filling factor, and the shape of holes in neighboring rows. The technological part of work revealed, that it is challenging to maintain the variable filling factor geometry during fiber drawing, when a demanding soft glass is used, although the developed structure is stable over the lengths of single-digit meters, while for supercontinuum generation only tens of centimeters are required. In the results reported herein, the agreement between the properties of fibers designed and of fibers actually drawn was unsatisfactory. Hence, additional methods of fiber structure control, e.g., variable pressure applied to the individual holes of the preform [33,34,35,36], should be considered.

## Figures and Tables

**Table 1:** Properties of selected glasses used for development of PCFs [5,13,22,23,24,25].

	Fused Silica	ZBLAN	Chalcogenide	Tellurite	Lead Silicate	PBG81
Linear refractive index *n*	1.44	1.51	2.45	2.08	1.80	1.89
Nonlinear refractive index *n*_2_ [10^−20^ m^2^ W^−1^]	4.3	2.1	250	51.1	8.9–22	41.3
Transmittance range [μm]	0.2–2.8	0.2–8	0.9–12	0.5–5	0.4–4.5	0.4–5.5
Zero dispersion wavelength [μm]	1.27	1.72	4.9	2.22	1.96	2.03

**Table 2:** Sellmeier’s equation coefficients for PBG-08A glass.

*B*_1_	*B*_2_	*B*_3_	*C*_1_	*C*_2_	*C*_3_
2.211153	0.355174	1.16141	0.01834	0.073012	127.3884

**Table 3:** Geometrical parameters of the dispersion-optimized PCF.

Parameter	Value
*Λ*	1.50 μm
*d*_1_	0.57 μm
*d*_1_/*Λ*	0.38
*d*_2_	0.96 μm
*d*_2_/*Λ*	0.64
*d*_3_	0.84 μm
*d*_3_/*Λ*	0.56
*d_r_*	2.43 μm

**Table 4:** Geometrical parameters of the selected developed PCFs.

	#A2	#A4
*Ø*_out_ [μm]	134	123
*Λ* [μm]	~1.8	~1.3
*d*_1_ [μm]	0.73	0.38
*d*_2_ [μm]	2.00 × 1.35	1.19 × 1.03
*d*_3_ [μm]	1.43	0.83
*d*_1_/*Λ*	0.52	0.32
*d*_2_/*Λ*	0.74	0.75
*d*_3_/*Λ*	0.80	0.64
*d_r_* [μm]	2.13	2.06
cladding size [μm]	33.5 × 29.5	24.5 × 20.9

**Table 5:** Measured ZDWs and dispersion values at intended pump wavelength for the fibers #A2 and #A4.

	#A2	#A4
*λ*_ZDW_ [µm]	1.326	1.525
*D_p_* @1.56 µm [ps/(nm·km)]	77.6	5.4

**Table 6:** Parameters of SG for the dispersion-optimized PCF.

*L_D_* [m]	*L_NL_* [mm]	*L_sol_* [mm]	*N*
44.4	0.39	133	334

**Table 7:** Nonlinear parameters for computer simulations in the fibers #A2 and #A4.

	*β*_2_ [ps^2^/km]	*β*_3_ [ps^3^/km]	γ [km^−1^ W^−1^]	L_D_ [m]	*L_NL_* [mm]	*L_sol_* [mm]	*N*
#A2	−61.5	0.42	557	2.6	0.30	27.9	93
#A4	25.1	0.23	468	6.4	0.36	47.6	134

**Table 8:** Width of the supercontinuum spectrum for the fibers #A2 and #A4.

	Dynamics 20 dB			Dynamics 30 dB		
	*λ_min_* [μm]	*λ_max_* [μm]	Δ*λ* [μm]	*λ_min_* [μm]	*λ_max_* [μm]	Δ*λ* [μm]
#A2	1.45	2.40	0.95	0.76	2.40	1.64
#A4	0.90	2.40	1.50	0.86	2.40	1.54
